# Sexual and reproductive health self-care interventions in the Eastern Mediterranean Region: findings from a cross-sectional values and preferences survey to inform WHO normative guidance on self-care interventions

**DOI:** 10.1186/s12961-020-00659-w

**Published:** 2021-04-21

**Authors:** Carmen H. Logie, Heather Abela, Tarek Turk, Samantha Parker, Karima Gholbzouri

**Affiliations:** 1 Factor Inwentash Faculty of Social Work, University of Toronto, 246 Bloor Street, Toronto, ON M5S 1V4 Canada; 2 Women’s College Research Institute, Women’s College Hospital, Toronto, Canada; 3 Department of Dermatology and Venereology, Damascus Hospital, Ministry of Health, Damascus, Syria; 4 Department of Psychiatry, Faculty of Medicine and Dentistry, University of Alberta, Edmonton, Canada; 5 Women’s Reproductive Health, WHO, EMRO, Cairo, Egypt

**Keywords:** Sexual and reproductive health, Attitudes, Eastern Mediterranean Region, Mobile health, Condom use, Health care provider training

## Abstract

**Background:**

Self-care strategies for sexual and reproductive health (SRH) include practices, tools, and strategies for people to manage their health. Access to SRH services has increased in the Eastern Mediterranean Region (EMR) in the past decade. The objective of this manuscript is to provide a preliminary assessment of self-care SRH interventions focusing on access, knowledge, perceived challenges, and recommendations for the future. We aim to contribute to the evidence base on knowledge and uptake of self-care SRH strategies in the EMR.

**Methods:**

We conducted an online cross-sectional Global Values and Preferences Survey (GVPS) to inform WHO guideline development on self-care interventions for SRH. Recruitment was web-based and included hosting the survey on the WHO Department of Reproductive Health and Research website, and sharing the survey link to diverse SRH websites. Analyses included the subsample of respondents living in EMR countries. We first conducted descriptive statistics of sociodemographic and self-care intervention responses. We then conducted bivariate analyses to examine statistically significant differences in knowledge for each intervention between EMR and non-EMR regions. We extracted open-text responses and applied thematic analysis techniques.

**Results:**

There were 53 respondents from the EMR spanning 14 countries, including16 health care providers (HCP) and 37 laypersons. Qualitative responses (*n* = 16) suggest that (a) perceived benefits of self-care SRH strategies include enhanced SRH access, knowledge, and improved SRH outcomes; (b) perceived concerns include misuse and safety; (c) linkage to care following self-care SRH interventions can consider mobile phone apps, hotlines, health care liaisons, and community outreach; (d) HCP want additional training on strengthening therapeutic alliances with patients and practical information on interventions; and (e) future research can focus on reproductive health, condom use, service barriers, and implementation. EMR respondents reported lower knowledge levels than non-EMR respondents on the following strategies: diaphragm/cervical cap, contraceptive patch, web-based SRH information, post-exposure prophylaxis, re-exposure prophylaxis, and HIV treatment.

**Conclusions:**

Knowledge of self-care SRH strategies varies by intervention type in the EMR. Future research with larger and more representative samples can inform regional self-care SRH implementation. Knowledge dissemination, stigma reduction, accessibility, and training of health care professionals are key domains for advancing access to self-care SRH strategies in the EMR.

## Background

Self-care strategies offer increased access, autonomy, and reduced stigma for advancing sexual and reproductive health (SRH) [1]. Self-care interventions include practices, tools, and strategies for people to take an active role in managing their own health. At their core, self-care interventions are underpinned by people- and systems-centred approaches. In the sphere of SRH, self-care strategies fall under the umbrellas of self-management (such as self-treatment, self-injection, self-medication, self-administration), self-testing (including self-testing, self-sampling), and self-awareness (such as self-help, self-education, self-efficacy) [1]. The WHO developed global normative guidance in 2019 on self-care interventions for sexual and reproductive health and rights (SRHR). The guidance emphasizes the importance of understanding places of access, the enabling environment, and accountability in order for self-care strategies to advance health, gender equality, and human rights across the life course [2]. While some self-care strategies are widely known and available across different global settings, such as condoms, others may be less acknowledged, available, and accessible, such as abortion self-management or self-injectable long-acting contraceptives [3].

Access to, and availability of, SRH services has increased in the Eastern Mediterranean Region (EMR) in the past decade [4]. For instance, access to and use of contraception is on the rise, and sexually transmitted infection (STI) voluntary counselling and testing programmes are reported in Lebanon, Algeria, Morocco, Jordan, and the Islamic Republic of Iran [5, 6]. The Islamic Republic of Iran and Morocco have HIV self-testing policies in place, and Afghanistan, Libya, Somalia, and Sudan have policies in development [7, 8]. While the overall HIV prevalence in the EMR remains low, there was a 10% increase in new cases between 2010 and 2018 [9]. At the population level there is a general high level of HIV knowledge in the EMR. However, certain populations, including women, sex workers, and people who use illicit drugs, may have lower levels of HIV knowledge than the general population [10].

Even where self-care policies are in place, such as in Pakistan and Qatar, knowledge gaps in provision of SRH counselling and education may lead to misuse of diagnostic tests or incorrect medication dosage [11, 12]. Stigma surrounding access to SRH services and sexually active unmarried persons presents barriers to SRH services access [5, 13]. The stigma toward people living with HIV is a persistent concern [5, 9, 14]. These intersecting forms of HIV and SRH stigma may be amplified for young persons [15]. While postnatal care, family planning, and breast cancer screening are available at the primary health care level [6], these services are developed for married women and may exclude young people due to stigma surrounding nonmarital sex [5]. Stigma surrounding SRH and sexual activity signal the salience of self-care strategies to advance SRH in the EMR.

Another phenomenon that highlights the relevance of self-care for SRHR to the EMR is the presence of conflict. With more than 16 million persons of concern, including 10.3 million internally displaced persons, 2.7 million refugees, and 2.3 million refugee and internally displaced returnees, the United Nations High Commissioner for Refugees (UNHCR) describes the Middle East and North Africa region as “the epicentre of global displacement challenges” (p. 100) [16]. Self-care interventions for SRHR have been described as particularly relevant for humanitarian and conflict-affected contexts that may have limited health care resources and insufficient health infrastructure, and may not have widespread evidence-based SRH policies, practices, and resources [17]. In light of scarce government resources, increased access to self-care SRH strategies may enhance access for all populations within conflict-affected countries [4]. Despite their relevance, there are knowledge gaps regarding SRH self-care access and priorities among forcibly displaced persons, including persons who may experience intersecting forms of stigma such as adolescents [17].

Self-care SRH interventions have been identified as priority areas for the EMR [11, 18]. However, only some nations in the region have established policies or services in this field. In addition, there is a scarcity of studies that have investigated perspectives from the EMR on self-care SRH strategies, or knowledge and perceived challenges among health care providers (HCPs). The manuscript objective is to provide a preliminary assessment focusing on access, knowledge, perceived challenges, and future recommendations regarding self-care strategies for the EMR. Our research questions included the following: (1) In the EMR, what is the knowledge of self-care SRH strategies and where to access them? (2) What are preferred places to access self-care SRH products and information? (3) Are there differences in knowledge of self-care SRH products between EMR and non-EMR regions? (4) What are perspectives from lay persons and HCPs in the EMR on self-care interventions for SRH?

## Methods

### Study design

We conducted a web-based cross-sectional survey between July 2018 and November 2018. This survey was developed following a series of expert WHO consultations to inform WHO’s consolidated guideline on self-care interventions for SRHR [2]. We received research ethics board approval from the University of Toronto, Canada. Details about the study and a full report of the methods and results can be found in the WHO Global Values and Preferences Survey (GVPS) report [19].

### Participant eligibility and recruitment

The study population included any adult capable of providing informed consent, including persons not involved in health care (we refer to as “lay persons”) as well as any cadre of HCPs. We aimed to include lay person perspectives to inform a people-centred framing of implementation practices for self-care for SRHR [1], and to engage HCP perspectives in order to elicit information to inform future guidelines, trainings, and practice briefs. Understanding both lay person and HCP perspectives aligns with Narasimhan, Allotey, and Hardon’s (2019) conceptual framework for self-care interventions that places the person practicing self-care at the centre of a system that includes an enabling environment with a trained health workforce [1]. Participant eligibility criteria included age of 18 years old or older, ability to read and comprehend a survey language (English, French, Spanish), and ability to provide web-based informed consent. We used two-web based strategies to recruit participants. The first was hosting the survey on the WHO Department of Reproductive Health and Research website, and the second was sharing a link (> 35) to the survey of diverse SRH websites (for a full list, see [3]). Our target sample size was 1000; for chi-square test of independence analyses with 18 degrees of freedom (17 self-care strategies and region), effect size = 0.20, power = 0.95, *p* < 0.05, the required sample size is 741.

### Survey procedures

This web-based survey aimed to elicit information on lay person (non-HCP) and HCP perspectives, knowledge, and uptake of a range of SRH self-care interventions relevant to the guideline. The survey was pilot-tested with three WHO self-care for SRHR Guideline Development Group members, and suggested modifications were integrated. The survey was hosted on the Qualtrics platform and included web-based informed consent as a prerequisite for completing the survey. The survey took approximately 20 minutes to complete. To minimize response bias, all questions were optional, and the surveys were anonymous, with no identifying information collected from participants. There were questions regarding self-care intervention knowledge, uptake, and information that were asked of all survey respondents. There was also a question on whether the respondent identified as a HCP; HCP-identified respondents had additional questions they could complete.

The survey questions addressed self-care interventions spanning reproductive health (oral contraceptive pill, emergency contraception, contraceptive patch, vaginal ring, self-injectable long-acting contraceptive, diaphragm/cervical cap, abortion self-management, web-based reproductive health information, reproductive health mobile application [app]) and sexual health (STI self-testing, HIV self-testing, post-exposure prophylaxis [PEP], pre-exposure prophylaxis [PrEP], HIV treatment [antiretroviral therapy], STI treatment, web-based sexual health information, sexual health mobile app). To assess knowledge we asked “have you heard of” for each intervention (responses dichotomized into yes [“I know what this is, and I know where to access”] versus no [I don’t know what this is, or I know what it is but I don’t know how to access]). To assess uptake we asked whether respondents or their partners had ever used this intervention (dichotomized into yes [ever having used, including in the past 3 months] versus no [including no, never having used and no, not viewing it as relevant]). To assess places of access, we asked what place they would prefer to access each intervention (responses: pharmacy, doctor/health clinic, online, I don’t know where to get it, I don’t need to use/not relevant) and where they would prefer to access information on that intervention (responses: online, friends and community, doctor/HCPs, I haven’t gotten any information on this).

We also included five open-text boxes to collect qualitative responses. Two of these questions were open to all participants: “Is there anything you would like to share with us about self-initiated interventions?”, and “Any other comments?”. The following open-text boxes were asked of HCP respondents: “What specific training, information or skills would you like regarding self-initiated interventions?”, “How do you think we can help to link patients/clients to health care if needed after using a self-initiated intervention?”, and “Are there any other benefits you can see regarding self-initiated interventions?”.

### Data analysis

For the analyses in this manuscript, out of all of the survey participants, we included only the subsample of respondents who identified their country of residence as an EMR country as defined by WHO. We first conducted descriptive statistics of the sociodemographic and self-care intervention responses. We then conducted bivariate analyses using chi-square tests of independence to examine statistically significant (*p* < 0.05) differences in knowledge (knowledge defined as knowing what the intervention was and how to access it) for each intervention by region (EMR versus non-EMR). We extracted open-text responses from Qualtrics into Microsoft Excel, and applied thematic analyses [20, 21] to evaluate the responses. Thematic analysis involves carefully reviewing the data and applying codes to generate inductive and deductive themes. Deductive codes were generated from the self-care SRHR conceptual framework [1], for instance components of an enabling environment for self-care include factors such as education and information. Inductive codes were generated from the data, such as recommendations from HCP regarding self-care strategies for SRHR. Two persons (CL, HA) coded each response, and codes were collated into thematic maps. Guideline development group members and experts on SRHR in the EMR (including manuscript co-authors KG and TT) reviewed the survey findings and provided feedback on analysis and interpretation to ensure content validity. Further information on methods, data analysis, and findings across geographical regions can be found in the WHO GVPS report [19].

## Results

There were 53 respondents (mean age: 30.44 years) from the EMR spanning 14 countries (see Table 1). Most participants identified as women (*n* = 32; 61.5%) and university-educated, including holding a bachelor’s degree (*n* = 8, 26.7%) or a graduate degree (*n* = 18, 60.0%). Out of 30 participants who responded to the question of whether they identified as a HCP, 16 (*n* = 53.3%) identified as HCP; the rest (*n* = 14; 46.7%) did not, and are referred to as lay persons in this manuscript.

**Table 1:** Respondent sociodemographic characteristics for GVPS participants in the EMR (*n* = 53)

Sociodemographic characteristic	N* (%)
Gender	
Women	32 (61.5)
Men	20 (38.5)
Total	52 (100.0)
Age categories	
18–29	32 (61.5)
30–39	6 (11.5)
40–49	9 (17.3)
50–59	4 (7.7)
60–69	1 (1.9)
70+	0 (0)
Total	52 (100.0)
*Mean*	*30.44*
*Median*	*24.00*
*Standard deviation*	*11.84*
*Range*	*20–63*
Subregion	
Eastern Africa	1 (1.9)
Northern Africa	23 (43.4)
Southern Asia	17 (32.1)
Western Asia	12 (22.6)
Total	53 (100.0)
Country	
Afghanistan	2 (3.8)
Egypt	3 (5.7)
Iran	5 (9.4)
Jordan	1 (1.9)
Lebanon	4 (7.5)
Morocco	2 (3.8)
Oman	2 (3.8)
Pakistan	10 (18.9)
Qatar	3 (5.7)
Somalia	1 (1.9)
Sudan	3 (5.7)
Syrian Arab Republic	1 (1.9)
Tunisia	15 (28.3)
Yemen	1 (1.9)
Total	53 (100.0)
Highest level of education	
None	0 (0)
Completed primary school	0 (0)
Completed high school	4 (13.3)
A university bachelor's degree	8 (26.7)
A graduate degree	18 (60.0)
Other	0 (0)
Total	30 (100.0)
HCP, educator or researcher	
Yes	16 (53.3)
No	14 (46.7)
Total	30 (100.0)

*Questions were optional, so total respondents varied per question

### Open-ended qualitative responses

Sixteen respondents (12 HCP and 4 lay persons) provided open-ended qualitative survey responses. Qualitative responses to the open-ended survey questions suggest that (a) perceived benefits include enhanced SRH access and knowledge and improved SRH outcomes; (b) perceived concerns include misuse and safety; (c) linkage to care following self-care SRH interventions can consider mobile phone apps, hotlines, health care liaisons, and community outreach; (d) HCP wanted additional training on establishing and strengthening therapeutic alliances with patients, communication skills, and more practical information on self-care SRH interventions; and (e) future research and practice recommendations included increased attention to reproductive health, condom use, context-specific service barriers, and implementation considerations to increase access.

### Perceived benefits and concerns regarding self-care interventions for SRH

When responding to the question regarding the benefits of self-care interventions for SRH, participants reported that self-injectable contraceptives would increase access and reduce mortality, and recommended that comprehensive information and quality control be provided with this intervention: “availability and use of self-injectable contraceptives can save millions of lives; however, this requires providing full information and availability of quality products” (lay person, Pakistan).

Provider perspectives included that a benefit of self-care interventions was to “raise knowledge” (HCP, Sudan). Others linked this increased knowledge to improved sexual health outcomes, describing that benefits of self-care interventions included “raising awareness and limiting sexually transmitted infections” (HCP, Egypt).

Some HCP expressed concerns regarding integrating self-care interventions into health systems. For instance, a HCP concern included misuse of contraceptives due to poor awareness of testing and diagnosis: “Misuse of various contraceptive methods and an almost total absence of awareness of tests and diagnostics” (HCP, Tunisia). Others described that patients’ safety was a concern: “an intervention led by a doctor or health care provider is safer and maintains safer reproductive health” (HCP, Pakistan).

### Recommendations for linkage to care after using SRH self-care strategies

HCPs shared a range of suggestions for linking persons to health care systems following the use of self-care SRH strategies. Several participants discussed mobile phone apps: “phone application or a site dedicated for this” (HCP, Tunisia). Others suggested a hotline: “Maybe through a hot line, home visits, a transportation to take the client to the service site” (HCP, Yemen), or a combination of both apps and hotlines (HCP, Sudan). Some noted the need for hotline confidentiality: “establish an easy way to contact, such as a hotline number for any inquiries, also providing a very high level of security and privacy” (HCP, Egypt).

Other suggestions were in-person methods with direct links to health care, such as “scheduled consultations for specific and supportive services” (HCP, Tunisia). Others discussed a health care liaison: “a guide to connect them to a relevant health care provider institution” (HCP, Pakistan). Developing community-level information sessions was another suggestion: “They need education using outreach workshops and symposiums” (HCP, Qatar).

### HCP perspectives on training, information, and skills they would like for self-care interventions

Several HCPs provided suggestions for training and skills regarding strengthening the therapeutic alliance between HCPs and patients. For instance, a HCP from Egypt recommended more training on “the power of trust and contact between the health care provider and the patients”. Others described wanting training on communicating with patients about stigmatized topics, such as sex: “how to explain to the patients the administration while avoiding embarrassing them, because the one that is related to sex is taboo for some” (HCP, Tunisia).

Knowledge dissemination on self-care interventions was another topic identified by HCP participants. This included specific information on self-care strategies: “about different types of interventions, side-effects, dealing with problems initiated from any of the type” (HCP, Yemen). Others highlighted the importance of accessing such information online: “SRH information on sites that are authentic and registered” (HCP, Pakistan). Several responded to the question about their self-care SRH training needs by noting that they wanted “practical techniques” (HCP, Sudan).

### Research and practice recommendations on self-care SRH interventions

When asked for suggestions about future research on self-care SRH strategies, many respondents requested that additional research focus on reproductive health. For instance, a HCP critiqued the survey for not focusing more on pregnancy outcomes or partner responses to sexual health challenges:

This survey should have been more in depth, categorizing different age groups, i.e. newly married, married for five to ten years, more years to specify, whether active in reproduction, number of pregnancies, number of safe deliveries, any miscarriage or abortion experienced, response of partner or spouse in case of any mishap related to sexual health. (HCP, Pakistan)

Condom use was identified as another area to include in future studies: “I noticed that condoms were not mentioned” (HCP, Yemen). Others identified gaps in their region: “I live in Jordan and unfortunately I haven’t heard of self-initiated HIV testing. No access. This is sad.” (HCP, Jordan). When asked whether they wanted to share any other considerations on self-care interventions, a participant described mistreatment when trying to access emergency contraception:

I have once been kicked out of a pharmacy for requesting an emergency contraceptive pill. On the other hand, there are a few health clinics funded by UN agencies that help us get the services we need but they still judge and interfere in our personal decisions and give advices. (lay person, Syrian Arab Republic)

Others described the need for research to study “cost-effectiveness” (HCP, Egypt). Some noted that strategies for SRH self-care should be developed to increase access: “[strategies should] be easy to access for all, not too costly, and simple to understand for the uneducated people” (HCP, Egypt). Participants also pointed out the need for more information on SRH self-care products, requesting “availability of full information and quality of the products” (layperson, Pakistan).

### Survey responses

#### Knowledge and uptake of self-care interventions for SRH

Table 2 reports the results of persons who knew what each self-care intervention was and how to access it, and those who had used the intervention themselves or their partner had. There was high variability in knowledge by self-care intervention. While the overwhelming majority knew about reproductive health interventions such as the oral contraceptive pill (98.0%) and emergency contraception (81.3%), fewer knew about self-injectable long-acting contraceptives (53.2%) or the diaphragm/cervical cap (45.7%). When it came to using reproductive health interventions, the strategies with the greatest number of respondents reporting having ever used were reproductive health information found online (51.2%), reproductive health mobile phone app (43.8%), emergency contraception (20.9%), and the oral contraceptive pill (18.6%). The strategies least used included the vaginal ring (2.4%) and the diaphragm/cervical cap (4.7%). Most participants reported knowing what abortion self-management was (70.8%), and one-tenth reported ever using this intervention (9.5%).

**Table 2:** Knowledge and uptake of SRH self-care interventions among GVPS participants in the EMR

Self-care intervention	I know what this is and where to go to access this *n* (%)	Myself/my partner ever used *n* (%)
*Reproductive health*		
Oral contraceptive pill	50 (98.0), total *n* = 51	8 (18.6), total *n* = 43
Emergency contraception	39 (81.3), total *n* = 48	9 (20.9), total *n* = 43
Contraceptive patch	26 (54.2), total *n* = 48	3 (7.0), total *n* = 43
Vaginal ring	29 (61.7), total *n* = 47	1 (2.4), total *n* = 42
Self-injectable long-acting contraceptive	25 (53.2), total *n* = 47	3 (7.1), total *n* = 42
Diaphragm or cervical cap	21 (45.7), total *n* = 46	2 (4.7), total *n* = 43
Abortion self-management	34 (70.8), total *n* = 48	4 (9.5), total *n* = 42
Web-based reproductive health information	35 (72.9), total *n* = 48	22 (51.2), total *n* = 43
Reproductive health mobile phone app	21 (43.8), total *n* = 48	10 (23.8), total *n* = 42
*Sexual health*		
STI self-testing	23 (47.9), total *n* = 48	4 (9.5), total *n* = 42
HIV self-testing	24 (50.0), total *n* = 48	5 (11.9), total *n* = 42
Post-exposure prophylaxis (PEP)	19 (40.4), total *n* = 47	3 (7.3), total *n* = 41
Pre-exposure prophylaxis (PrEP)	17 (37.0), total *n* = 46	4 (9.5), total *n* = 42
HIV treatment (antiretroviral therapy)	32 (66.7), total *n* = 48	0 (0.0), total *n* = 42
STI medication treatment	34 (75.6), total *n* = 45	4 (9.5), total *n* = 42
Web-based sexual health information	36 (75.0), total *n* = 48	20 (47.6), total *n* = 42
Sexual health mobile phone app	11 (22.9), total *n* = 48	7 (16.7), total *n* = 42

With regard to sexual health interventions, the greatest knowledge pertained to STI treatment (75.6%), HIV treatment/antiretroviral therapy (66.7%), and HIV self-testing (50.0%). Participants reported the lowest knowledge about PrEP (37.0%) and PEP (40.4%).

#### Places of access to self-care SRH products and information

In Table 3 we report places where respondents reported accessing each intervention. Sources of access varied greatly by product. For instance, the top three products most likely to be accessed at a pharmacy were the oral contraceptive pill (50.0%), emergency contraception (41.7%), and the contraceptive patch (28.6%). The most frequently accessed product from a doctor or health clinic was STI treatment (50.0%). Online access was reported for web-based sexual (44.1%) and reproductive (41.2%) health information, reproductive health apps (37.1%), and sexual health apps (34.3%). The products that respondents most frequently reported not knowing where to access were PEP (30.3%), PrEP (27.3%), and sexual health apps (25.7%).

**Table 3:** Access to SRH self-care interventions among GVPS participants in the EMR

Self-care intervention	Access: places						Access: informational sources				
	Pharmacy *n* (%)	Doctor or health clinic *n* (%)	Would buy it online *n* (%)	I don’t know where to get it *n* (%)	I don’t need to use this (not relevant) *n* (%)	Total respondents *n*	I go online / internet *n* (%)	I ask my friends and community *n* (%)	I ask a doctor or health care provider *n* (%)	I haven’t gotten any information on this *n* (%)	Total respondents *n*
*Reproductive health*											
Oral contraceptive pill	19 (50.0)	18 (47.4)	0 (0.0)	0 (0.0)	9 (23.7)	38	9 (36.0)	6 (24.0)	17 (68.0)	2 (8.0)	25
Emergency contraception	15 (41.7)	17 (47.2)	0 (0.0)	0 (0.0)	8 (22.2)	36	11 (47.8)	4 (17.4)	11 (47.8)	2 (8.7)	23
Contraceptive patch	10 (28.6)	15 (42.9)	0 (0.0)	5 (14.3)	8 (22.9)	35	6 (26.1)	4 (17.4)	15 (65.2)	3 (13.0)	23
Vaginal ring	6 (17.6)	15 (44.1)	0 (0.0)	6 (17.6)	9 (26.5)	34	4 (18.2)	2 (9.1)	15 (68.2)	4 (18.2)	22
Self-injectable long-acting contraceptive	5 (14.7)	15 (44.1)	0 (0.0)	6 (17.6)	10 (29.4)	34	5 (22.7)	3 (13.6)	15 (68.2)	4 (18.2)	22
Diaphragm or cervical cap	6 (18.2)	13 (39.4)	1 (3.0)	5 (15.2)	11 (33.3)	33	5 (22.7)	2 (9.1)	15 (68.2)	4 (18.2)	22
Abortion self-management	5 (14.7)	16 (47.1)	2 (5.9)	3 (8.8)	11 (32.4)	34	7 (31.8)	4 (18.2)	15 (68.2)	2 (9.1)	22
Web-based reproductive health information	2 (5.9)	10 (29.4)	14 (41.2)	4 (11.8)	4 (11.8)	34	14 (58.3)	2 (8.3)	12 (50.0)	2 (8.3)	24
Reproductive health mobile phone app	1 (2.9)	9 (25.7)	13 (37.1)	8 (22.9)	6 (17.1)	35	13 (56.5)	2 (8.7)	8 (34.8)	4 (17.4)	23
*Sexual health*											
STI self-testing	3 (8.8)	15 (44.1)	1 (2.9)	6 (17.6)	11 (32.4)	34	6 (27.3)	1 (4.5)	15 (68.2)	6 (27.3)	22
HIV self-testing	3 (8.8)	15 (44.1)	1 (2.9)	6 (17.6)	12 (35.3)	34	8 (34.8)	2 (8.7)	15 (65.2)	6 (26.1)	23
Post-exposure prophylaxis (PEP)	2 (6.1)	12 (36.4)	0 (0.0)	10 (30.3)	12 (36.4)	33	7 (30.4)	0 (0.0)	15 (65.2)	5 (21.7)	23
Pre-exposure prophylaxis (PrEP)	4 (12.1)	14 (42.4)	0 (0.0)	9 (27.3)	12 (36.4)	33	6 (27.3)	0 (0.0)	13 (59.1)	5 (22.7)	22
HIV treatment (antiretroviral therapy)	1 (2.9)	16 (47.1)	2 (5.9)	4 (11.8)	13 (38.2)	34	7 (30.4)	0 (0.0)	16 (69.6)	4 (17.4)	23
STI medication treatment	3 (8.8)	17 (50.0)	0 (0.0)	5 (14.7)	12 (35.3)	34	8 (34.8)	1 (4.3)	1 (4.3)	2 (8.7)	23
Web-based sexual health information	1 (2.9)	10 (29.4)	15 (44.1)	4 (11.8)	6 (17.6)	34	14 (58.3)	1 (4.2)	13 (54.2)	1 (4.2)	24
Sexual health mobile phone app	1 (2.9)	9 (25.7)	12 (34.3)	9 (25.7)	7 (20.0)	35	13 (54.2)	2 (8.3)	8 (33.3)	5 (20.8)	24

When seeking information on self-care SRH products, the internet was most commonly used to access information on emergency contraception (47.8%) as well as SRH web-based information and apps. People were most likely to ask their friends and community about the oral contraceptive pill (24.0%) and abortion self-management (18.2%). Doctors and HCPs were commonly used sources of information for most self-care SRH interventions, including HIV treatment (69.6%) and a range of contraceptive options (vaginal ring, self-injectable long-acting contraceptive, diaphragm/cervical cap, abortion self-management, all at 68.2%). The products that respondents were most likely to report not having received any information on included STI self-testing (27.3%), HIV self-testing (26.1%), and PrEP (22.7%).

#### Knowledge of self-care interventions for SRH in EMR and non-EMR regions

Table 4 reports findings from comparative analysis between knowledge of self-care SRH strategies for EMR versus non-EMR country respondents. Reproductive health strategies where knowledge was statistically significantly lower at the *p* < 0.05 level for EMR respondents than non-EMR respondents included the diaphragm/cervical cap, contraceptive patch, and web-based reproductive health information. Sexual health interventions that were statistically significantly lower at the *p* < 0.05 level for EMR respondents than non-EMR respondents included web-based sexual health information, sexual health apps, PEP, HIV treatment/antiretroviral therapy, and PrEP.

**Table 4:** Knowledge of self-care SRH interventions among GVPS participants by EMR residence

Self-care intervention	Yes, I know what this is and where to go to access this		Chi-square
	EMR *n* (%)	Non-EMR *n* (%)	
*Reproductive health*			
Oral contraceptive pill	50 (98.0), total *n* = 51	698 (95.4), total *n* = 732	2.681, *p*-value 0.262
Emergency contraception	39 (81.3), total *n* = 48	653 (89.2), total *n* = 732	3.039, *p*-value 0.219
Contraceptive patch	**26 (54.2), total n = 48**	**516 (70.9), total n = 728**	23.345, p-value 0.000**
Vaginal ring	29 (61.7), total *n* = 47	474 (64.9), total *n* = 730	1.692, *p*-value 0.429
Self-injectable long-acting contraceptive	25 (53.2), total *n* = 47	461 (63.4), total *n* = 727	2.342, *p*-value 0.310
Diaphragm or cervical cap	**21 (45.7), total n = 46**	**502 (69.3), total n = 724**	28.106, p-value 0.000**
Abortion self-management	34 (70.8), total *n* = 48	439 (60.2), total *n* = 729	5.038, *p*-value 0.081
Web-based reproductive health information	**35 (72.9), total n = 48**	**658 (90.4), total n = 728**	22.209, p-value 0.000**
Reproductive health mobile phone app	21 (43.8), total *n* = 48	404 (55.6), total *n* = 727	3.559, *p*-value 0.169
*Sexual health*	23 (47.9), total *n* = 48	337 (46.4), total *n* = 727	0.703, *p*-value 0.704
STI self-testing	24 (50.0), total *n* = 48	383 (52.8), total *n* = 726	0.174, *p*-value 0.917
HIV self-testing			
Post-exposure prophylaxis (PEP)	**19 (40.4), total n = 47**	**444 (61.2), total n = 725**	8.613, p-value 0.013*
Pre-exposure prophylaxis (PrEP)	**17 (37.0), total n = 46**	**412 (57.1), total n = 721**	7.264, p-value 0.026*
HIV treatment (antiretroviral therapy)	**32 (66.7), total n = 48**	**593 (81.6), total n = 727**	8.169, p-value 0.017*
STI medication treatment	34 (75.6), total *n* = 45	571 (79.0), total *n* = 723	3.119, *p*-value 0.210
Web-based sexual health information	**36 (75.0), total n = 48**	**648 (89.1), total n = 727**	10.903, p-value 0.004**
Sexual health mobile phone app	**11 (22.9), total n = 48**	**334 (45.7), total n = 731**	9.652, p-value 0.008**

*p < 0.05; **p < 0.01

## Discussion

This study provides insight into self-care SRH interventions among a small sample of lay persons and HCP in the EMR. We found a range of knowledge of SRH interventions, varying by type of intervention. Aligned with the literature [5], most participants were knowledgeable about oral contraceptives, which in turn was the most commonly used contraceptive choice reported by participants. As emergency contraception was used even more frequently than the oral contraceptive pill, heath systems can further examine barriers to oral contraceptive use and widening contraceptive choices. Web-based reproductive health information and mobile apps were also commonly used, indicating the reach of mobile health (mHealth) in the EMR. Most participants reported knowledge of STI and HIV treatments, and half were aware of HIV self-testing. Important differences in knowledge between EMR and non-EMR regions include that regarding PEP and PrEP—EMR participants were the least likely to know about these two interventions.

The biggest knowledge gaps pertained to self-injectable long-acting contraceptives and the diaphragm/cervical cap. Compared to non-EMR regions, respondents in the EMR had significantly less knowledge of the diaphragm/cervical cap and contraceptive patch. This provides actionable information for policy-makers and HCPs to expand the range of contraceptive options. While participants in EMR countries reported high knowledge of web-based reproductive health information compared to other SRH self-care interventions, it was still lower than that in non-EMR regions. This was true for sexual health information accessed online and sexual health mobile apps. Barriers to mHealth could be further explored in the EMR, for instance, paying close attention to languages that these services are offered in, and cultural and contextual relevance. Knowledge of HIV treatment was also significantly lower in the EMR versus non-EMR, and this could also be associated with HIV-related stigma, whereby lower knowledge of HIV can contribute to increased stigma. Increasing comprehensive sexuality education could both increase HIV knowledge and reduce HIV stigma.

The open-ended qualitative responses expand our understanding of the quantitative survey data by signalling the need to facilitate linkages to care, with many innovative suggestions provided including hotlines, apps, and community outreach alongside self-care SRH interventions. Qualitative findings highlight improved SRH access, knowledge, and outcomes as benefits of self-care strategies, with some safety concerns. HCPs identified two distinct types of training needs: the first was communication and building a therapeutic alliance with patients—including ways to approach and discuss stigmatized topics—and the second was self-care intervention-specific information. Suggested areas for future research included reproductive health, condoms, and strategies to enhance access to both information and products.

There were several notable study limitations. These included non-random sampling and language barriers, as the survey was not conducted in Arabic: these limit the generalizability of findings. The sample was also highly educated, so there was likely a bias toward including persons already knowledgeable regarding SRH and self-care strategies. The small sample size of EMR participants precluded country-specific analyses. As recruitment was conducted online, we included persons more likely to have access to internet and computer resources who were familiar with the WHO website and SRH listservs. A larger survey in Arabic with diverse recruitment strategies is needed to elicit more representative perspectives and understandings of self-care interventions for SRH in the EMR. However, this GVPS [19] is the largest to date on knowledge of self-care SRH interventions, and produced a global snapshot of self-care for SRH that allowed us to both focus on EMR-specific responses and to compare knowledge between EMR and non-EMR regions. Despite these limitations, this manuscript provides an initial scoping endeavour in this field of self-care for SRH for an EMR population and can inform and direct future research.

## Conclusion

There are varying levels of knowledge regarding self-care strategies for SRH in the EMR. More studies are needed to inform intervention implementation strategies. For instance, research could explore HIV self-testing delivery preferences, such as web-based ordering or pharmacy pickup. Mixed-methods research could examine barriers and facilitators to PrEP uptake, and examine preferences in administration (such as oral versus injectable) as well as adherence support needs. Stigma-focused research could explore community perspectives on various self-care strategies for SRHR, paying particular attention to stigma drivers such as misinformation, and stigma facilitators such as inequitable gender norms, in order to inform stigma reduction interventions [22]. Knowledge dissemination, stigma reduction, accessibility, and training of health care professionals are important domains to investigate to advance access to self-care SRH interventions. This might be particularly relevant to marginalized and conflict-affected populations in the EMR, as the potential for self-care to enhance their SRH is underexplored. Yet self-care interventions have particular relevance for advancing SRH among forcibly displaced persons [17]. For instance, humanitarian settings often lack sufficiently trained health care workers, have inadequate SRH infrastructure, and may lack comprehensive, private, and confidential SRH practices [17]. These barriers experienced by forcibly displaced persons may be, at least in part, reduced through increased options to achieve SRH through self-care strategies. Further research on values, preferences, feasibility, and priorities of self-care strategies in humanitarian contexts is warranted [17], particularly during the COVID-19 pandemic, when people may experience heightened barriers to SRH care [23]. mHealth seems to be a promising avenue for increasing accessibility to self-care in the EMR and warrants further investigation.

## Data Availability

Available upon request.

## Abbreviations

EMR: Eastern Mediterranean Region
GVPS: Global Values and Preferences Survey
HCP: Health care providers
PEP: Post-exposure prophylaxis
PrEP: Pre-exposure prophylaxis
SRH: Sexual and reproductive health
SRHR: Sexual and reproductive health and rights
STI: Sexually transmitted infections
UNHCR: United Nations High Commissioner for Refugees
WHO: World Health Organization
